# The Celluloid Infringement

**Published:** 1880-12

**Authors:** 


*The Celluloid Infringement.*—The Supreme Court of the United States has confirmed the decision of the Supreme Court of the District of Columbia, that the use of celluloid is not an infringement of the patent owned by the Goodyear Dental Vulcanite Company. This ends the struggle of the giant monopoly, which for years has sought to absorb everything in the reach of its money and its attorneys. The end of its exactions draws near, however, as the patent expires in May, 1881, when, unless they license dentists under the "Hæring" patent, as it is claimed they intend, the ubiquitous rubber agent will no longer visit the dental practitioners. Of all the men who have worked hard without pay to fight the claims of this company, none deserve more honorable mention than Dr. R. Finley Hunt, of Washington, D. C., who gave the subject many months of hard toil, both of brain and hand, and who worked up against the Rubber Company the most complete presentation of worthless patents and of casuistry and unfairness in dealing with a profession that has ever been recorded. Dr. Hunt deserves the thanks of all who are in any way concerned in seeing a monopoly broken, or an injustice continued in perpetration.

